# Supplementary material for: Risk Models for Breast Cancer and Their Validation
Source: Stat Sci. Author manuscript; Available in PMC 2020 Mar 27. (PMC7100774; doi:10.1214/19-STS729)
Supplement: Supplementary figures [file EMS85958-supplement-Supplementary_figures.pdf]

Supplementary material for ‘Risk models for breast cancer and their validation’

Adam R Brentnall, Jack Cuzick

Centre for Cancer Prevention, Wolfson Institute of Preventive Medicine, Queen Mary University of London, Charterhouse square, London, EC1M 6BQ e-mail: `a.brentnall@qmul.ac.uk`*;* `j.cuzick@qmul.ac.uk`*.*

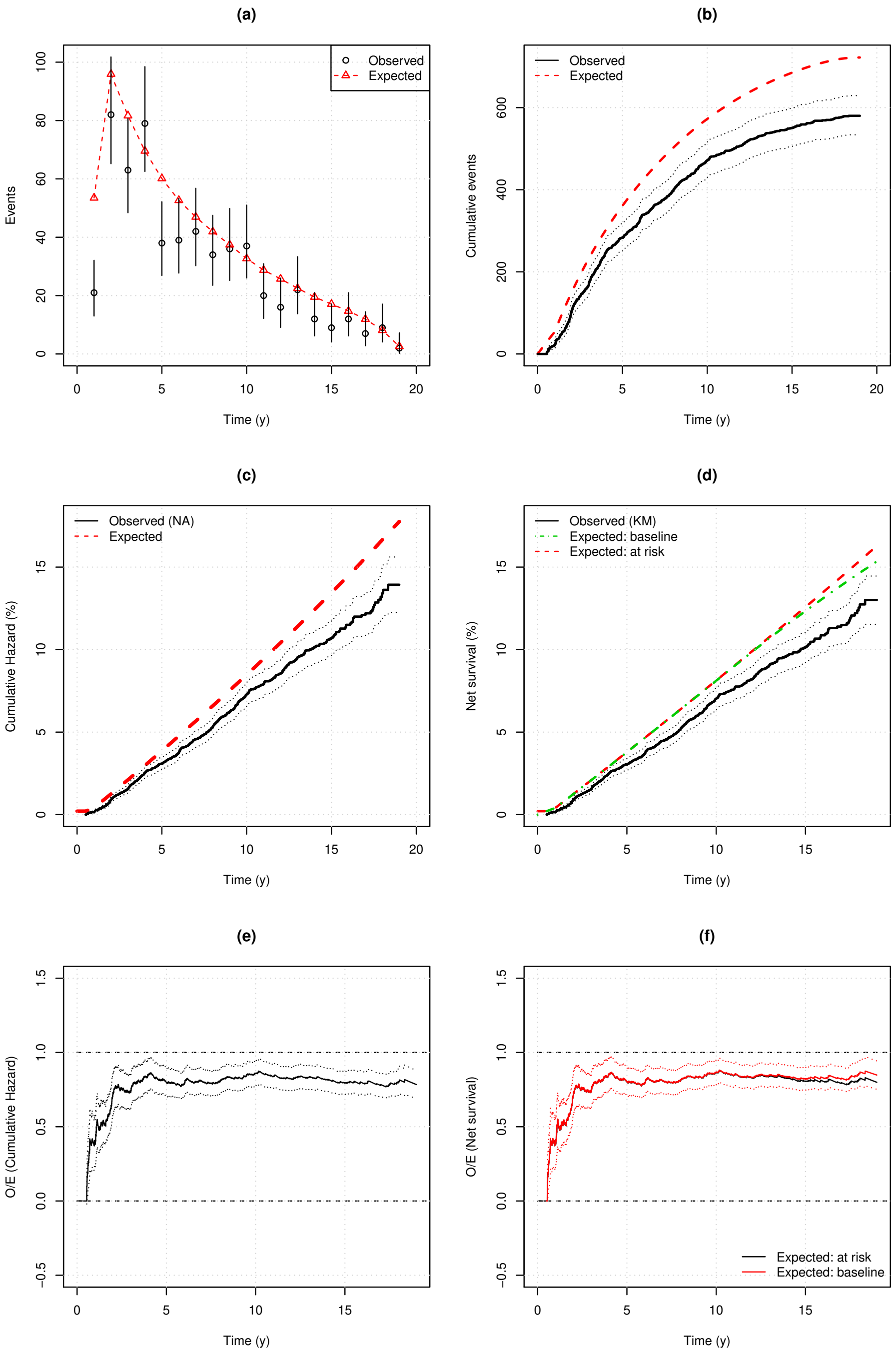

FIG S1. *Calibration of top decile of 10y predicted risk (>5.4% 10y net risk). (a) Observed (95%CI) vs expected number of breast cancers diagnosed for each year of follow-up, (b) cumulative observed (95%CI) vs expected number of breast cancers diagnosed; (c) observed (Nelson-Aalan, 95%CI) vs expected cumulative hazards; (d) observed (Kaplan-Meier, 95%CI) vs expected (obtained via two methods) net risks; (e) Observed divided by Expected cumulative hazard (95%CI); (f) Observed divided by expected net risk (obtained via two methods) with 95%CI only for the expected risk based on baseline risk assessment.*

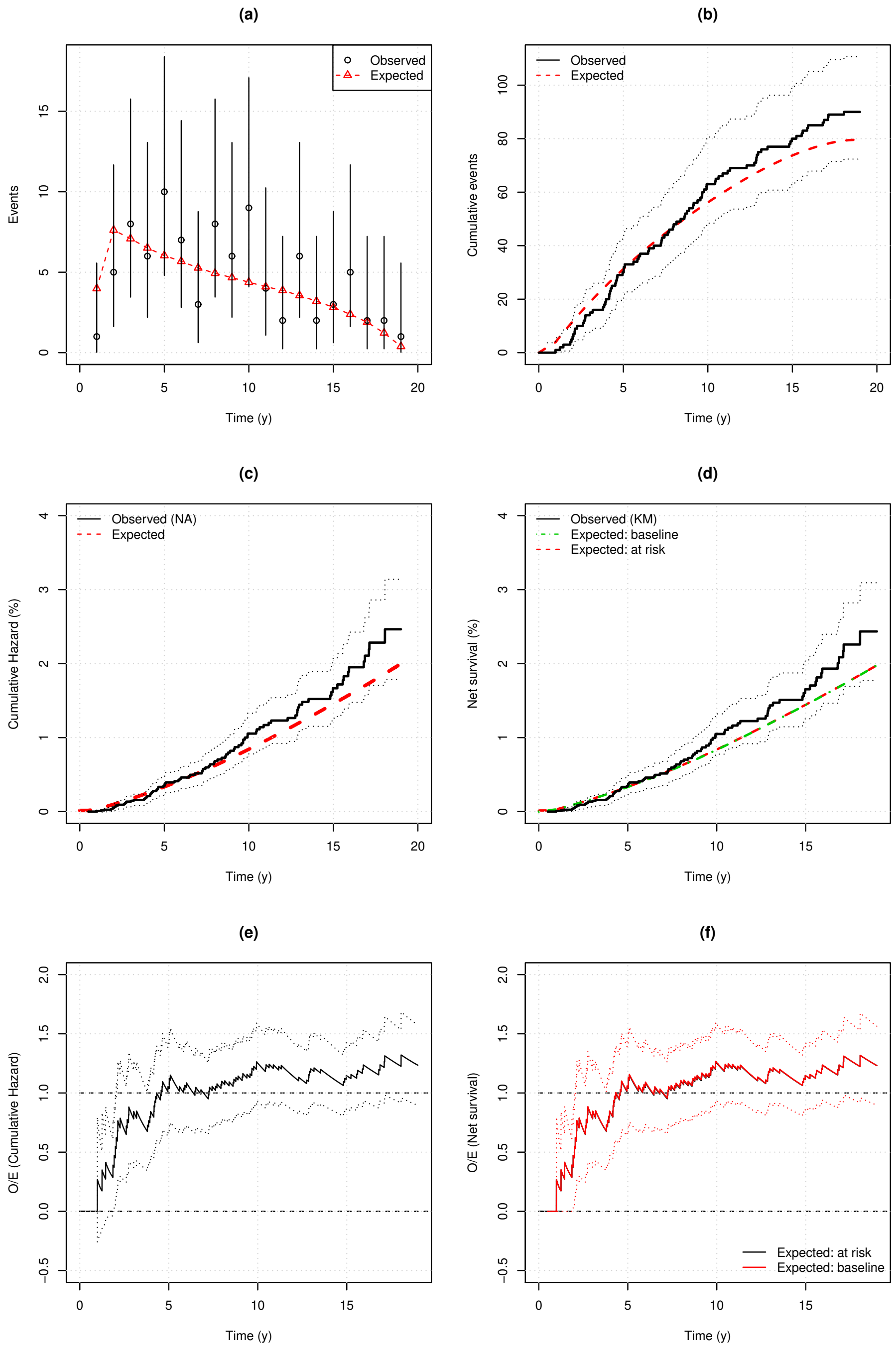

Fig S2. *Calibration of bottom decile of 10y predicted risk (<1.1% 10y net risk). (a) Observed (95%CI) vs expected number of breast cancers diagnosed for each year of follow-up, (b) cumulative observed (95%CI) vs expected number of breast cancers diagnosed; (c) observed (Nelson-Aalan, 95%CI) vs expected cumulative hazards; (d) observed (Kaplan-Meier, 95%CI) vs expected (obtained via two methods) net risks; (e) Observed divided by Expected cumulative hazard (95%CI); (f) Observed divided by expected net risk (obtained via two methods) with 95%CI only for the expected risk based on baseline risk assessment.*
